# Harnessing nature’s arsenal: sustainable plant-based strategies for phytopathogen control

**DOI:** 10.3389/fmicb.2025.1588462

**Published:** 2025-06-18

**Authors:** Gabriel G. Calefi, Nagela B. S. Silva, Bader Y. Alhatlani, Emad M. Abdallah, Carlos H. G. Martins

**Affiliations:** ^1^Laboratory of Antimicrobial Testing, Institute of Biomedical Sciences (ICBIM), Federal University of Uberlândia, Uberlândia, Brazil; ^2^Unit of Scientific Research, Applied College, Qassim University, Buraydah, Saudi Arabia; ^3^Department of Biology, College of Science, Qassim University, Buraydah, Saudi Arabia

**Keywords:** plant extract, phytopathogens, economic impact, chemical composition, antimicrobial activity

## Abstract

Phytopathogens represent a persistent threat to global agricultural productivity, precipitating yield losses and destabilizing food security. Conventional reliance on synthetic agrochemicals, while effective in phytopathogen suppression, incurs significant economic burdens, drives environmental toxicity, and accelerates the evolution of resistant microbial strains, with collateral risks to ecosystem integrity and public health. This review synthesizes current advancements in harnessing plant- and microorganism-derived extracts, bioactivity-guided fractions, and purified phytochemicals as eco-compatible antimicrobial agents against phytopathogenic bacteria and fungi. Furthermore, we propose a novel framework for standardized prioritization of natural products, integrating efficacy thresholds, phytochemical complexity, and mechanistic specificity to guide scalable antimicrobial discovery. Meta-analysis of published studies reveals a predominant focus on Fusarium spp. as model phytopathogens, with dilution in broth and agar diffusion as the predominant *in vitro* assays. Quantitative benchmarks for antimicrobial potential were established: bacterial Minimum Inhibitory Concentrations (MICs) ≤ 2.5 mg/mL (crude extracts), ≤0.6 mg/mL (fractions), and ≤64 μg/mL (purified compounds), alongside fungal growth inhibition thresholds <52% (agar dilution assays). These criteria highlight the differential bioactivity of natural product tiers, emphasizing the role of compound purification in potency enhancement. By bridging phytochemical innovation with agronomic applicability, this work positions plant-derived antimicrobials as pivotal tools for sustainable disease management, circumventing agrochemical limitations while advancing One Health-aligned agricultural practices.

## 1 Introduction

As the global population nears >9 billion by 2050, humanity faces a critical paradox: persistent hunger and caloric insufficiency coexist with rising overnutrition and obesity (Guldan, 2020). This dual burden of malnutrition strains health systems, exacerbates socioeconomic inequalities, and necessitates innovative, equitable solutions to achieve sustainable food security through strategic agricultural investment (Ulian et al., 2020). Plant cultivation has played a foundational role in agricultural systems since the advent of human civilization, serving as a critical driver of food security and socioeconomic development with substantial economic value. Nevertheless, crop productivity and quality remain persistently threatened by microbial pathogens including viruses, bacteria, fungi and oomycetes that compromise plant health and yield. Notable phytopathogens include bacteria such as *Pseudomonas viridiflava*, *Escherichia coli*, *Xanthomonas campestris*, *Bacillus megaterium*, and *Clavibacter michiganensis*; fungi such as *Aspergillus micheli*, *Alternaria alternata*, *Fusarium oxysporum*, *Penicillium digitatum*, and *Botrytis cinerea*; oomycetes such as *Phytophthora cinnamomi*, *Pythium aphanidermatum*, and *Phytophthora infestans*; and plant viruses such as Dasheen mosaic virus (DMV), Sour cherry green ring mottle virus (CGRMV), and Potato leafroll virus (PLRV). These diverse pathogens are responsible for significant agricultural losses (Elshafie et al., 2021; Giachero et al., 2022; Hartman, 1974; Hernández-Díaz et al., 2021; Martin and Loper, 1999; Taliansky et al., 2003; Zhang et al., 1998). For instance, the grey mould fungus, *Botrytis cinerea*, is a significant contributor to pre- and post-harvest losses in fruit and vegetable production. Recently classified as a ‘high-risk’ necrotrophic pathogen, it exhibits a remarkable capacity to rapidly develop resistance to fungicides, primarily through drug efflux transport mechanisms (Weber and Petridis, 2023; Shao et al., 2021). Of particular concern, intensive and excessive fungicide applications have led to the emergence of multiresistant strains in several countries, posing a serious challenge to disease management strategies (Hahn, 2014).

Contemporary agricultural practices for pathogen management remain heavily reliant on synthetic agrochemicals. However, conventional pest control strategies frequently fail to account for their broader ecological and economic ramifications. Prolonged agrochemical use has been associated with adverse environmental impacts, including bioaccumulation within trophic networks, resulting in biomagnification of toxic compounds across food chains (Dhananjayan et al., 2020). Human health risks, such as acute intoxication and chronic poisoning, further underscore the limitations of these chemical agents (Devi et al., 2022; Lekei et al., 2014). Moreover, the indiscriminate application of agrochemicals accelerates the evolution of antimicrobial resistance, wherein phytopathogenic strains acquire adaptive mechanisms to circumvent chemical control measures. This necessitates the deployment of increasingly potent compounds, perpetuating a cycle of environmental degradation and ecological imbalance (Lekei et al., 2014). In response to these challenges, the scientific community has intensified efforts to identify sustainable alternatives that harmonize economic viability with ecological safety. Natural products, encompassing bioactive compounds, phytochemical fractions, and plant-derived nanoparticles, have emerged as promising candidates due to their biodegradability, low environmental persistence, and reduced propensity for resistance development (Chin et al., 2006). By leveraging these resources, researchers aim to mitigate the unintended consequences of industrial agrochemicals while maintaining robust antimicrobial efficacy.

However, significant research gaps hinder their translation from lab to field. First, while *in vitro* studies demonstrate efficacy, mechanistic insights into how plant-based compounds such as Olive mill wastewater (rich in phenolics) interact with Phytopathogenic bacteria like *Pseudomonas savastanoi* pv. *savastanoi, Clavibacter michiganensis,* and *Xanthomonas campestris* remain limited, impeding optimization (Košćak et al., 2023). Second, the yield of bioactive compounds extracted from a given plant is highly variable due to the influence of climatic conditions and many other factors. This inconsistency is further compounded by the absence of standardized extraction protocols for isolating phytochemicals with potential activity against phytopathogenic microorganisms, posing a significant challenge to their reliable application in plant disease management (Bitwell et al., 2023; Kumar et al., 2017; Moomin et al., 2023). Third, the potential of synergistic combinations between plant-derived compounds and biocontrol organisms or integrated phytopathogen management strategies remains largely unexplored. Despite their promise in enhancing the effectiveness and sustainability of phytopathogen control, such approaches have received limited research attention and, when applied, are largely restricted to greenhouse crops (Pandit et al., 2022). Fourth, long-term ecological impacts—such as effects on non-target species or soil microbiomes—are poorly documented, raising questions about holistic sustainability (Nadeu et al., 2023). Finally, economic barriers, including cost–benefit analyses and adoption incentives, are overlooked in favor of purely technical research, and limiting real-world uptake. This review critically examines recent advances in the application of natural products derived from plants as antiphytopathogenic agents, with a focus on their mechanistic action and efficacy. We further evaluate standardized methodologies for antimicrobial assessment and propose a unified criterion for interpreting antibacterial and antifungal activity data, aiming to establish a framework for identifying high-potency, environmentally sustainable phytopathogen control strategies.

## 2 Major phytopathogens

The agroecosystem plays a pivotal role in shaping local and global economies. However, agricultural productivity is frequently compromised by phytopathogens, leading to significant yield reductions and economic losses. Among viral pathogens, *mosaic viruses* (e.g., *Tobacco mosaic virus*) are particularly impactful (Palukaitis et al., 1992). Predominant fungal phytopathogens include genera such as *Aspergillus*, *Fusarium*, *Penicillium*, *Alternaria*, and *Botrytis*, which infect diverse plant tissues, including leaves, stems, roots, and fruits (Nakajima and Akutsu, 2014; Okungbowa and Shittu, 2012; Peever et al., 2002). Bacterial genera such as *Ralstonia*, *Pseudomonas*, *Xanthomonas*, *Pectobacterium*, and *Dickeya*, similarly can colonize and damage plants through either systemic or localized infections (Álvarez et al., 2010; Bashan and De-Bashan, 2002; Ge et al., 2021; Rudolph, 1993). Table 1 summarizes the most prevalent fungal and bacterial phytopathogenic genera and their associated host crops.

**Table 1:** Major genera of bacterial, fungal, oomycetes and viruses phytopathogens and their affected plants and fruits.

Biological group	Phytopathogens	Crops affected	Reference
Bacteria	*Clavibacter* spp.	Tomato, alfalfa, wheat, potato, bean	Nandi et al. (2018) and Peritore-Galve et al. (2021)
	*Dickeya* spp.	Potato	Toth et al. (2011)
	*Erwinia* spp.	Ornamental plants, apples, pear and potatoes	Reiter et al. (2002) and Vrancken et al. (2013)
	*Pectobacterium* spp.	Potato, tomato, maize, cabbage, and ornamental plants, rice, maize, potato	Ma et al. (2007), Toth et al. (2021), and Werra et al. (2021)
	*Pseudomonas* spp.	A*rabidopsis* sp., sweet basil	Mansfield et al. (2012) and Walker et al. (2004)
	*Ralstonia* spp.	Potato, tomato, and other solanaceous plant species	Pedro-Jove et al. (2021)
	*Xanthomonas* spp.	*Arabidopsis thaliana,* broccoli, cabbage, cauliflower, citrus fruit	Papaianni et al. (2020) and Reynol (2017)
	*Xylella* spp.	Grape, olive, almond, citrus fruit, coffee	Bruening et al. (2014) and Landa et al. (2022)
Fungi	*Alternaria* spp.	Tomato, olive, carrots, citrus fruits, cereals, apples	Logrieco et al. (2009) and Palou et al. (2012)
	*Aspergillus* spp.	Maize, cottonseed, peanuts, tree nuts	Diener et al. (1987) and Robens and Cardwell (2003)
	*Botrytis* spp.	Strawberry, citrus fruits, grapes, chickpea, and other beans	Boddy (2016) and Pande et al. (2006)
	*Colletotrichum* spp.	Woody ornamentals and tropical foliage plants, papaya, mango, guava, avocado	Azevedo Dos Santos et al. (2020)
	*Fusarium* spp.	Wheat, soft red winter wheat, durum wheat, barley, bananas	Dita et al. (2018), Nelson (1992), and Nganje et al. (2004)
	*Glomerella* sp.	Apple	Wang et al. (2015)
	*Penicillium* spp.	Apple, garlic	Wang et al. (2023)
	*Rhizoctonia* spp.	Soybean, corn, alfalfa, winter wheat, spring barley	Malvick (2018) and Smiley (2021)
	*Rhizopus* spp.	Sunflower, strawberry, passion fruit	Schipper and Stalpers (1984) and Thompson et al. (1980)
Oomycetes	*Aphanomyces* spp.	Pea, sugar beet, spinach alfalfa, lentils, green beans	Wikström et al. (2025)
	*Peronospora* sp.	Alfalfa	Yu et al. (2023)
	*Phytophthora* spp.	Pepper, cucumber, pumpkin, tomatoes, snapbeans	Granke et al. (2012)
	*Pythium* spp.	Avocado, pineapple, peach, chestnut, macadamia, camellia, oak, pine and eucalyptus	Hardham (2005) and Jung et al. (2013)
Virus	*Nepovirus*	Cucumber, Blueberries	Caglayan et al. (2025) and James et al. (2024)
	Polerovirus	Tobacco, wild-rice	Yan et al. (2025)
	Potyvirus	Potato	Mäkinen et al. (2023)

Phytopathogens encompasses a diverse spectrum of pathogens, including viroids, viruses, prokaryotic bacteria, and eukaryotic organisms such as fungi, oomycetes, and nematodes (Pandit et al., 2022). The following sections provide an overview of the major phytopathogenic fungi, bacteria, and selected viral pathogens, highlighting the diseases they cause and their economic impact on agricultural production systems. Particular attention is given to viral diseases in a dedicated section.

### 2.1 Fungi phytopathogens

Fungal phytopathogens play an important role in global food and health security issues, causing various diseases in many crops and fruits (Luck et al., 2011). Fungi such as *Aspergillus* are mainly found in soil, fruits, plants and have more than two hundred species such as *A. terreus, A. fumigatus* and *A. oryzae.* This genus produces conidiophores, a structure similar to the cnidarian “hydra,” which allows it to reproduce and spread (Jayaprakash et al., 2019). The disease caused by these fungi is mainly due to toxins such as aflatoxin (Amaike and Keller, 2011), a type of mycotoxin, which affects various stages of plant life, such as seed germination and other physiological processes, causing harm to humans and animals when ingested in contaminated foods (Abdelaziz et al., 2022). According to Eskola et al. (2020), between 60 and 80% of all crops in the world are damaged by mycotoxins such as aflatoxicins.

In the United States, maize cultivation suffers losses of approximately US$160 million per year due to aflatoxin contamination. In developing countries, the impact is even more severe, reaching around US$450 million, which accounts for 38% of global losses caused by the presence of toxins in agricultural production (Jallow et al., 2021).

Another extremely important genus is *Fusarium*, which mainly infects the banana plant and fruit, and includes species such as: *F. oxysporum*, *F. sporotrichoides, F. graminearum* (Ploetz, 2015). Banana cultivation has a global market turnover of $25 billion dollars (Voora et al., 2020). India is the country with the largest banana production in the world, growing more than 31 million tons annually, surpassing China with an annual production of approximately 11 million tons and Indonesia with 8 million tons (Statista, 2024). *Fusarium* infection of the plant mainly affects the vascular system in the stem and root (Ploetz, 2015) and may reach the xylem (Gordon, 2017). It also has the potential to infect animals and humans, with a high mortality rate (Hof, 2020). *Fusarium oxysporum*, the primary agent of vascular wilt, stands out as the most widespread pathogen, with global crop losses estimated at 10–50%, and even higher in India, where losses can reach up to 80% (Bai et al., 2018).

The genus *Alternaria* includes the species: *A. alternata*, *A. arbusti, A. blumeae, A. brassicae, A. brassicicola, A. carotiincultae, A. conjuncta, A. dauci, A. euphorbiicola, A. infectoria, A. molesta, A. panax, A. petroselini, A. selini, A. solani, A. smyrnii,* among others, are known to attack pre- and post-harvest fruit, especially tomatoes (Palou et al., 2012). Infection by this fungus consists of the appearance of black spots on the skin of the fruit due to the production of toxins and diseases such as black mould rot (Yan et al., 2015). This genus can affect numerous plant species such as citrus fruits, pears, carrots, barley, oats, olives, melons, peppers, apples, raspberries, cranberries, grapes, sunflower seeds, melons, lentils, wheat and other grains (Crous et al., 2015; Logrieco et al., 2009; Patriarca et al., 2007). As recent research has shown (Shabeer et al., 2022), this type of genus can also infect humans and cause disease (Azcarate et al., 2008).

The *Botrytis* genus consists of 22 species such as *B. cinerea, B. convoluta, B. fabae*, among others that can cause the rust disease, characterized by leaf loss, black spot and decay in the stem (Bika et al., 2021). The most commonly affected plants are strawberries, citrus fruits, grapes, chickpeas and lettuce (Pande et al., 2006).

The *Glomerella* and *Colletotrichum* genera mainly attack apples, but they can also infect strawberries, causing leaf spots of *Glomerella* (Gonzalez et al., 2006) or even bitter rot, appearing as black or brown spots that grow and cause necrosis in the area (Moreira et al., 2019). They can also cause the disease anthracnose, causing rot on plants and dark lesions on fruit, affecting strawberry, mango, citrus fruit, avocado, banana, coffee and some cereal crops (Cannon et al., 2012). Both genera are related to each other, as the genus *Glomerella* represents the teleomorphic (sexual) phase of the fungus while the asexual (anamorphic) phase is represented by the genus *Colletotrichum* (Guerber and Correll, 2001), like the life cycle of cnidarians, alternating between polyp and medusa. A good example of a species that symbolizes this relationship is *G. cingulata*, which is the teleomorph of *C. cingulata*. *G. cingulata* in the epidemic regions of China caused severe damage to susceptible apples, including 90% defoliation and diseased fruit before harvest, which resulted in reduced tree vigor, lower yields and poor fruit quality (Liu et al., 2023).

The genus *Rhizoctonia* is made up of numerous species such as *R. fumigata, R. ferruginea, R. oryzae-sativae* and *R. rubi*, which reduce the yield of a large number of plants, from aquatic to aerial, such as soybeans, corn, alfalfa, winter wheat and spring barley (Malvick, 2018). It is most commonly found in damp soil, initiating the infection in the roots of the plant (Baker, 1970). *Rhizoctonia* bare spot affected almost 20% of the plantation (approximately 2 fields of 50 hectares), and the impact of the disease caused yield reductions of up to 73% on a winter wheat and spring barley farm (Smiley, 2021). Symptoms observed on a variety of hosts include seed decay, root decay, hypocotyl decay, crown decay, stem decay, limb and pod decay, stem canker, black scab and seedling blight (Ajayi-Oyetunde and Bradley, 2018).

The *Rhizopus* genus includes species such as *R. oryzae, R. nigricans* and *R. sexualis*, which infect sunflowers, strawberries, passion fruit and cause flower rot (SCHIPPER & STALPERS Schipper and Stalpers, 1984). The disease can be on the flower head or on the peduncle. On the flower, the disease manifests itself as a brown spot, causing some seeds to taste bitter and fall off. On the peduncle the whole flower falls off and rots (Yildirim et al., 2010).

Oomycetes are microorganisms that produce hyphae just like fungi. Their cell walls are composed of cellulose, and they produce coenocytic hyphae, which are not composed of divided cells but rather a single elongated cell (Hardham, 2007). In literature studies have been carried out on numerous oomycetes, such as the genera *Phytophthora* and *Pythium*. One of the most studied oomycetes in the literature is *Phytophthora*, especially *Phytophthora infestans*, which causes late blight in potatoes (Kamoun et al., 2015). The *Pythium* genera consist of *P. ultimum*, *P. aphanidermatum* and *P. irregulare*. These microorganisms cause diseases such as *Pythium* damping off, root rot (Mavrodi et al., 2012).

Chitosan, saponins, and induced systemic resistance play a crucial role in managing fungal and oomycete pathogens such as *Fusarium oxysporum*, *Phytophthora infestans*, and *Colletotrichum gloeosporioides*. It was reported that, chitosan interacts with the negatively charged components of the fungal cell surface, altering membrane permeability and leading to the leakage of intracellular electrolytes and protein-rich contents (Hernández-Lauzardo et al., 2011). Saponin-rich plant extracts from *Balanites aegyptiaca* fruit mesocarp, *Quillaja saponaria* bark, and *Yucca schidigera* have demonstrated effective inhibitory activity against phytopathogens such as *Fusarium oxysporum*, *Colletotrichum coccodes*, and *Verticillium dahliae*, with varying degrees of efficacy (Chapagain et al., 2007). Interestingly, systemic acquired resistance and induced systemic resistance are plant defense mechanisms triggered by prior infection or treatment, enhancing resistance to future pathogen attacks. Instead of directly targeting pathogens, they strengthen the plant’s physical and chemical barriers through signaling pathways, particularly salicylic acid-dependent cascades, that induce broad-spectrum, long-lasting resistance (Kamle et al., 2020).

### 2.2 Bacterial phytopathogens

Bacterial pathogens affect the global market for various fruits and vegetables commonly used in everyday cooking by the world’s population. A very relevant genus of phytopathogenic bacteria is *Ralstonia*, which usually infects the host through the roots, mainly affecting tomato plantations, responsible for bacterial wilt, a disease characterized mainly by the crumpling of leaves, eventually collapsing the plant (Tahat and Sijam, 2010). This genus includes species such as *R. pseudosolanacearum, R. solenacearum* and *R. syzygii*. To start the invasion, *R. solanacearum* initially releases enzymes that break down the cell wall of the host cell (Coll and Valls, 2013). The current widely accepted route of colonization by *R. solanacearum* involves the bacterium entering the root cortex of the host plant and subsequently progressing through the intercellular space to reach the xylem. Once in the xylem, the bacterium multiplies and spreads to the above-ground parts of the host plant (Bae et al., 2015).

Another extremely important disease in crops is soft rot, which affects the roots of plants such as potatoes, causing the tissue to decompose. The pathogens responsible include *Pectobacterium* and *Pseudomonas* spp. The genus *Pectobacterium* sp. is made up of species such as *P. carotovorum*, *P. atrosepticum*, *P. wasabiae*; and the genus *Pseudomonas* sp. includes *P. aeruginosa*, *P. putida*, *P. chlororaphis*. *Pectobacterium* sp., which can cause soft rot on various hosts, such as cabbage, carrots, celery, cotton, cucumbers, cyclamen, hyacinth, corn, potatoes, sugar cane and tobacco (Dye, 1968). Another disease is blackleg, which damages profits and agriculture in general. Symptoms include blackening of the stem and rotting (Van der Merwe et al., 2010). Infection begins when the bacterium comes into contact with the plant’s tissues through a wound, subsequently the tissue becomes soft due to the action of pectinolytic enzymes excreted by the pathogen, presenting a fetid secretion (Perombelon, 1988).

*Erwinia* includes species such as *Erwinia amylovora* and *Erwinia carotovora*. It is responsible for causing diseases such as blackleg of potatoes and fire blight (Reiter et al., 2002; Zeng et al., 2021). A 2024 study estimated the economic impact of fire blight on the orchard’s long-term economic performance, and, with the worst-case scenario, there was a reduction of up to 70% in the field’s profitability (Nieto et al., 2024).

*Xanthomonas* sp. is another genus of great importance in agriculture and includes species such as *X. citri, X. alfalfae*, *X. oryzae, X. vasicola* and *X. perforans*. These pathogens can cause circular, oily spots on leaves, stems, thorns and fruit and develop into white or yellow pustules that darken and thicken, forming a brown canker and deep craters in the fruit, which can lead to defoliation and premature fruit drop (Brunings and Gabriel, 2003). It is a genus of Gram-negative bacteria, which mainly affects citrus fruits such as lemons, oranges and tangerines (Starr, 1981). In 2019, according to the Fund for Citrus Protection (Fundecitrus 2019), in one region of Brazil, the incidence of symptomatic trees reached 15%, which is significant given the severity of the disease.

The genus *Dickeya* comprises bacteria that infect potato, tomato, chicory, artichoke, dahlia, kalanchoe, pineapple, sweet potato, banana, corn and others, including species such as *D. zeae, D. solani* and *D. aquatica*. The main symptom of infection caused by these bacteria is soft rot, which manifests itself as internal stem necrosis or rot extending from the base of the stem. Externally, however, the base of the stem appears healthy (Toth et al., 2011).

The *Clavibacter* genus includes Gram-positive bacteria such as *C. michiganensis, C. capsici,* and *C. sepedonicus*. During infection they can cause ratoon dwarfism, which is a term used to describe a problem prevalent in perennial crops such as sugar cane, sweet potato and certain varieties of banana. The term “ratoon” refers to the secondary plant growth that appears after the primary crop has been harvested, originating from the remaining shoots and buds of the original plants (Davis et al., 1984). One of the main species that causes major economic losses is *C. michiganensis*, as there have been reports of tomato fields losing between 50 and 100% of total production, and its symptoms are wide-ranging, such as cankers on the stem, dehydration of the leaf margins, chlorotic spots on the leaves, among others (Rossi, 2014).

**Figure 1:**
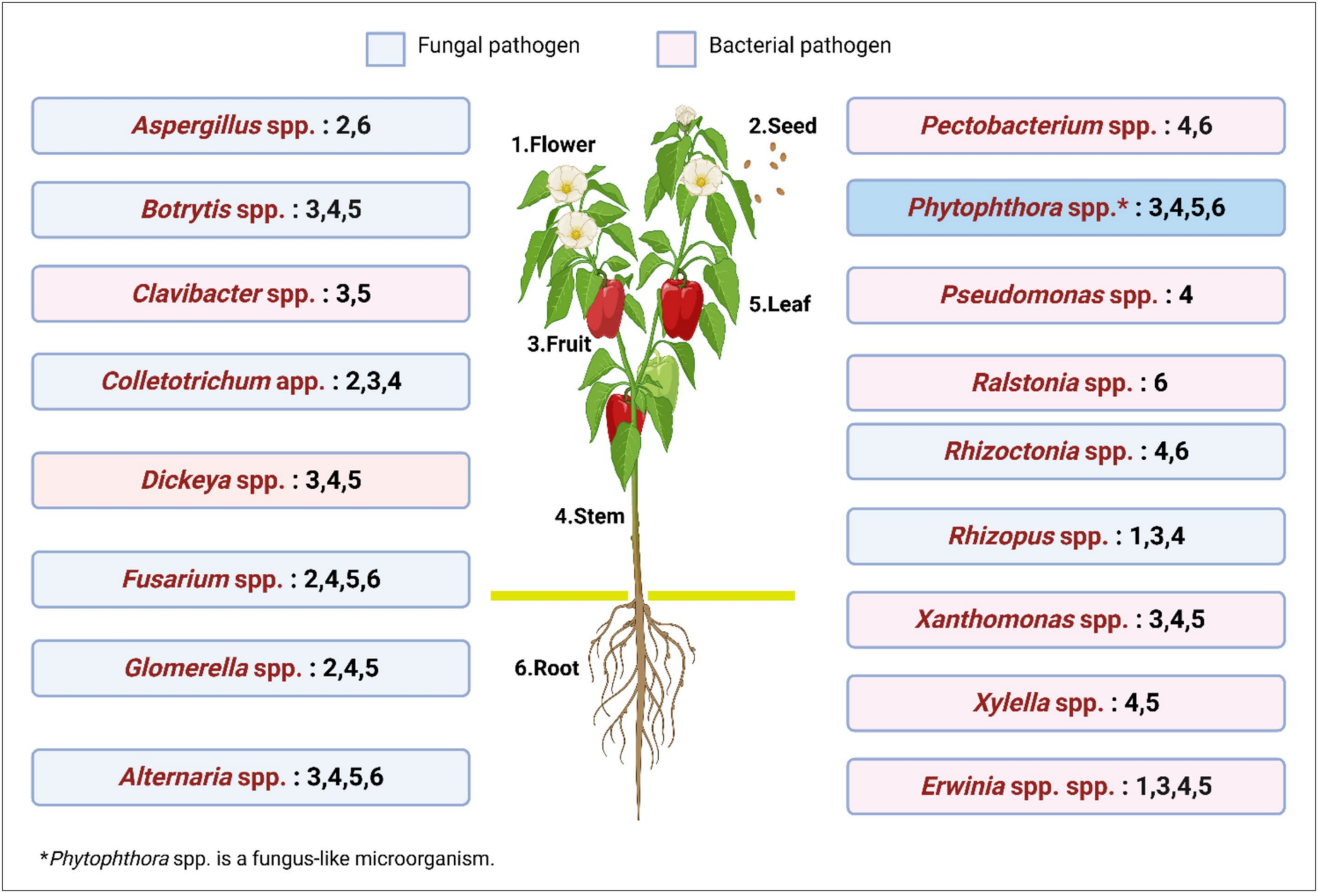
Key plant structures affected by common bacterial and fungal phytopathogens in agricultural plantations.

The *Xylella* genus can cause various plant diseases, such as citrus variegated chlorosis, plum leaf scald, false peach disease, olive rapid decline syndrome, Pierce’s grapevine disease, alfalfa dwarfing, margin necrosis and leaf scorch (Trkulja et al., 2022). The only member of its genus is *X. fastidiosa*, which is usually characterized by its subspecies as: *X. fastidiosa* subsp. *fastidiosa*, *X. fastidiosa* subsp. *pauca*, *X. fastidiosa* subsp. *multiplex*, *X. fastidiosa* subsp. *sandyi*, *X. fastidiosa* subsp. *morus*. Generally, these bacteria obstruct the transport of water and soluble minerals through the xylem, leading to various manifestations in infected plants, such as necrosis of leaf margins, wilting and subsequent drying of leaves and branches, along with stunted growth and wilting of specific parts of the plant. According to Schneider et al. (2020), the economic impact of ineffective control against *Xylella* subspecies would cause a batch in Italy to lose between US$2 billion and US$5.6 billion dollars (converted on 31/07/2024). Brazil, one of the higher production of citrus fruit, in 2015, had an orange production volume of 16.7 million tons (Pereira et al., 2022), and the country has been dealing with measures to protect orange and citrus production from citrus canker since 1957 (Behlau, 2021). Figure 1 illustrates the primary plant structures susceptible to infection by common bacterial and fungal phytopathogens in agricultural plantations. It highlights the specific sites of pathogen attack, providing insights into disease progression and potential impact on plant health.

Plant-derived antibacterial agents and microbiome manipulation represent sustainable strategies to combat bacterial phytopathogens. A variety of plant-derived phytochemicals (including extracts and essential oils) show potential as novel antibacterial agents for suppressing bacterial phytopathogens, offering sustainable alternatives to synthetic bactericides in agriculture (Abdullahi et al., 2020). A current trend in sustainable agriculture involves activating the soil microbiome by enhancing indigenous microbial communities, which has been shown to reduce diseases cause by phytopathogens such as bacterial wilt, brown blotch, fire blight, and crown gall. This approach offers effective plant protection while maintaining soil health, with no reported adverse effects on soil properties (Haq et al., 2024).

### 2.3 Viral phytopathogens

Plant viral diseases pose a significant challenge to global agriculture, reducing crop yield and quality. Besides, the increasing globalization of agriculture and international trade is contributing to the spread of viruses and their vectors into new regions, leading to unpredictable impacts on food production and natural ecosystems (Jones and Naidu, 2019). Unlike bacterial or fungal pathogens, plant viruses lack independent metabolic machinery and must rely on host cellular mechanisms for replication and systemic spread.

Plant viruses belong to various families with distinct transmission mechanisms, host ranges, and disease symptoms. The Potyviridae family includes *Potato Virus Y (PVY)*, which affects potatoes, tomatoes, and peppers, causing mosaic symptoms, leaf necrosis, and reduced yield (Scholthof et al., 2011). Another example is *Papaya Ringspot Virus* (PRSV), which infects papaya and cucurbits, leading to severe leaf distortion, mosaic patterns, and ring spots on fruit (Tripathi et al., 2008). The Tombusviridae family includes *Tomato Bushy Stunt Virus* (TBSV), which infects tomatoes and causes stunting, yellowing, and fruit malformations (Scholthof et al., 2011). *Cucumber Necrosis Virus* (CNV) is another member that affects cucumbers, causing necrotic lesions and stunted growth (Dijkstra and Khan, 2024). The Geminiviridae family includes *Tomato Yellow Leaf Curl Virus* (TYLCV), a devastating virus in tomatoes, transmitted by whiteflies (*Bemisia tabaci*), causing yellowing, curling of leaves, and severe yield loss (Navas-Castillo et al., 2011). Another example is *Maize Streak Virus* (MSV), which affects maize crops, causing chlorotic streaking and stunted growth (Bosque-Pérez, 2000).

Host resistance is a fundamental natural control mechanism. Some plants have evolved resistance genes (R genes) that recognize specific viral proteins and activate defense responses. RNA interference (RNAi) is another natural defense in which plants degrade viral RNA to prevent replication and movement (Baulcombe, 2004). Additionally, systemic acquired resistance (SAR) primes plants against secondary infections by inducing defense-related proteins and secondary metabolites (Durrant and Dong, 2004). Beneficial microorganisms, including endophytic fungi, rhizobacteria, and mycorrhizal fungi, enhance plant immune responses against viral infections. Certain strains of *Bacillus* and *Pseudomonas* produce antimicrobial compounds and elicit induced systemic resistance (ISR), reducing viral replication and symptom severity (Kloepper et al., 2004). Some fungal biocontrol agents, such as *Trichoderma* spp., secrete enzymes that degrade viral coat proteins, interfering with infection (Shoresh et al., 2010).

Many plant viruses are transmitted by insect vectors such as aphids, whiteflies, and thrips. Natural enemies like ladybugs (*Coccinellidae*), lacewings (*Chrysopidae*), and parasitoid wasps (*Encarsia formosa*) regulate vector populations, reducing virus transmission rates. Additionally, entomopathogenic fungi such as *Beauveria bassiana* and *Metarhizium anisopliae* target insect vectors and minimize virus spread (Baverstock et al., 2010). Certain plant extracts and essential oils exhibit antiviral activity by interfering with viral replication and cell-to-cell movement. Phytochemicals such as flavonoids, alkaloids, and terpenoids have shown inhibitory effects against plant viruses (Ma and Yao, 2020). Extracts from neem (*Azadirachta indica*) and garlic (*Allium sativum*) reduce infection severity by disrupting viral coat proteins and inhibiting vector feeding behavior (Thomas et al., 2021). Integrating natural control measures into agricultural systems requires a holistic approach. Crop rotation and intercropping disrupt virus life cycles and limit vector establishment. Companion planting with repellent species, such as marigold (*Tagetes* spp.) and basil (*Ocimum basilicum*), deters insect vectors and reduces virus incidence (Hooks and Fereres, 2006). Additionally, breeding programs focusing on durable resistance genes can provide long-term solutions against viral diseases (Garcia-Ruiz, 2018).

## 3 Plant-based alternatives for phytopathogen control: from extraction to evaluation

### 3.1 Extraction of bioactive plant compounds for phytopathogen control

It is evident that phytopathogens can simultaneously affect multiple plant structures. Consequently, advancements in microbial and pest control strategies continue to evolve annually, including the development of agrochemicals with enhanced antimicrobial efficacy, the utilization of natural biopesticides, and the induction of plant resistance to specific pests (Douglas, 2018; Perdikis et al., 2011; Vurro et al., 2019). The use of herbal medicines with various biological activities, such as antimicrobial, anti-inflammatory and anticarcinogenic, among others, is very wide and variable. There are different ways of extracting natural plant extracts, such as maceration, infusion, digestion, microwave-assisted extraction, percolation and steam distillation (Zhang et al., 2018). Maceration consists of crushing the leaves and can be done in a mortar and pestle (Ishii and Yokotsuka, 2014). Infusion is an extraction method that basically consists of pouring hot water on the leaves or part of the plant. Digestion is the decomposition of plant material with acids and high temperatures (Parr et al., 2001). Microwave-assisted extraction, as the name suggests, uses microwaves to heat the base material of the plant to obtain the extract (Kaufmann and Christen, 2002). The percolation method of solvent extraction consists of extracting the plant material by continuously flowing solvent through plant material to extract soluble components (Kaufmann and Christen, 2002; Singh, 2008), and steam distillation consists of applying steam directly to the plant material followed by condensation to obtain the extract (Cassel et al., 2009). In addition to the previously mentioned extraction techniques, advanced analytical methods such as High-Performance Liquid Chromatography (HPLC) and Gas Chromatography (GC) are widely utilized for the precise identification and quantification of bioactive compounds in plant-derived extracts.

Moussi et al. (2015) used the chromatography technique to separate the phenolic part of the *Rhamnus alaternus* L. extract, where due to interactions with the molecules of both the mobile part (solvent) and the stationary part (material filling the column), only the molecule or class of molecules of interest were separated. In the case of gas chromatography, separation depends on volatility and interaction with the stationary part (Bartle and Myers, 2002). The difference between liquid and gas is what carries the sample, which can be gas (helium, nitrogen or hydrogen) or liquid (Giddings, 1965). Moussi et al. (2015) employed High-Performance Liquid Chromatography (HPLC) to isolate phenolic compounds from *Rhamnus alaternus* L. extracts. In HPLC, separation is governed by differential interactions between analytes and two distinct phases: a liquid mobile phase (e.g., water, methanol, or acetonitrile) that transports the sample through the system, and a solid stationary phase (e.g., silica-based or polymer-packed columns) that selectively retains molecules based on polarity, size, or affinity (Snyder et al., 2011). For phenolic compounds, which are polar, non-volatile, and thermally labile molecules, HPLC is particularly advantageous, as it preserves their structural integrity while effectively resolving complex mixtures into individual components, such as flavonoids and phenolic acids, using gradient elution or isocratic modes (Moussi et al., 2015).

In contrast, Gas Chromatography (GC) utilizes a gaseous mobile phase (e.g., helium, nitrogen, or hydrogen) to transport volatilized analytes through a temperature-controlled column coated with a stationary phase (e.g., polysiloxane). Separation in GC depends primarily on volatility and secondarily on interactions with the stationary phase (Bartle and Myers, 2002). Compounds with lower boiling points elute faster, whereas polar or less volatile molecules often require derivatization, a chemical modification that enhances thermal stability and volatility, prior to analysis (Grob and Barry, 2004). While GC is ideal for volatile organic compounds (VOCs) such as terpenes and fatty acids, it is unsuitable for non-volatile phenolics, as these compounds would degrade under the high-temperature conditions of GC (Snyder et al., 2011). Thus, the choice between HPLC and GC hinges on the physicochemical properties of the analytes. HPLC excels in separating non-volatile, thermally sensitive molecules, including polyphenols and proteins, due to its gentle operating conditions and compatibility with polar solvents. Conversely, GC is reserved for volatile, thermally stable compounds such as hydrocarbons and essential oils, leveraging its ability to resolve low-molecular-weight species under elevated temperatures (Bartle and Myers, 2002; Grob and Barry, 2004).

A natural product extract is a concentrated preparation of active substances extracted from plants, vegetables, animals, bacteria and fungi derivatives. In several studies using natural extracts with antimicrobial activity, such as Pacheco-Cano et al. (2020), which used the crude extract of *Brassica oleracea* var. *italica* against *Bacillus cereus, Listeria monocytogenes, P. aeruginosa, Salmonella typhimurium* and *Vibrio parahaemolyticus,* and Fontana et al. (2022) witch evaluated the antimicrobial activity of the methanolic and hydroalcoholic extracts of *Moringa oleifera* against *Erwinia amylovora*. Compounds, on the other hand, consist of the material or chemical that has been separated from a mixture or source and obtained in its pure form, such as the isolation of a class of molecules or a specific molecule. Some studies have used compounds as Azevedo Dos Santos et al. (2020), who evaluated the peptide portion of *Capsicum chinense* fruit extract against *F. oxysporum*, *F. solani*, *Colletotrichum lindemuthianum* and *C. gloeosporioides*, Kim et al. (2018) who evaluated five antifungal molecules from the crude methanolic extract of *Trevesia palmat a* against *Alternaria porri, B. cinerea, Colletotrichum coccodes, F. oxysporum, Magnaporthe* oryzae and *Phytophthora infestans* showing promising results.

The literature contains articles describing the antimicrobial evaluation of natural extracts coated with nanoparticles, materials with dimensions in the nanometric range, usually between 1 and 100 nanometers (nm) (El-Naggar et al., 2024; Ndolomingo et al., 2020). Due to their very small size and high surface-to-volume ratio, nanoparticles exhibit distinct and unique properties Danish et al. (2022), with green synthesis for the production of silver nanoparticles based on the natural extract of *Cassia fistula* (L.), evaluated against *Pseudomonas syringae*, *Fusarium oxysporum*, *Rhizoctonia solani* and *Sarocladium* sp.

### 3.2 The search for evidence: microbiological evaluation of plant-based antimicrobials

To evaluate the biological potential of extracts, fractions and compounds from extracts or plant products, whether coated with nanoparticles, some techniques can be used, such as agar dilution, agar diffusion, micro and macro dilution in broth, bioautography, among others. When the method for antimicrobial evaluation is carried out on agar, techniques such as agar dilution or agar diffusion can be used. In literature, it is common to use different terms to describe the same methodological approach to assessing antifungal activity in filamentous fungi. Terms such as “poisoned food technique”(Pinto et al., 2022), “poisoned food medium assay”(Kim et al., 2019), “diffusion technique on PDA growth medium”(Vuerich et al., 2023), and “mycelial growth inhibition by an agar-dilution method”(Reyes et al., 2022), to describe methods in which the tested substance is incorporated into the agar before solidification. and then inoculating the hyphae-producing microorganisms, i.e., filamentous fungi on the agar plate, and the result is normally presented as the percentage of inhibition of the growth of the hyphae of the microorganism compared to a control without the sample (Perrucci et al., 1994; Balouiri et al., 2016).

Diffusion on agar, on the other hand, can be carried out in two main ways: disk or well on agar. In the case of agar well diffusion, as carried out by Bhatti et al. (2024), the microorganisms are inoculated onto the agar. Subsequently, a well is made in the agar to place the extracts or compounds (Magaldi et al., 2004). In the case of the paper disk, as performed by Klančnik et al. (2009), subsequently the agar plates are produced normally, sequentially the microorganism will be inoculated into the agar, and finally, paper discs containing the tested substance will be placed on the previously inoculated agar. In this way the tested samples will spread in part of the agar through diffusion (Magaldi et al., 2004). The test is interpreted by measuring the growth inhibition halo after the respective incubation of the microorganism used, thus making it possible to measure and account for the antimicrobial activity of the extracts and compounds (Devillers et al., 1989).

There are other methods such as: microdilution and macrodilution, which are characterized by serial dilution, and can be performed in tubes, in the case of macrodilution (Nikitin et al., 2023), or in wells in 96-well plates in the case of microdilution (Chalo et al., 2023). After diluting the sample, microorganisms are added at specific concentrations to determine the Minimum Inhibitory Concentration (MIC), defined as the concentration of the extract, or nanoparticle capable of compounds inhibiting the growth of the microorganism or eliminating it completely (Pa, 2002). The MIC can be interpreted by adding an oxidoreductive salt, such as rezasurin, which changes color from blue to pink when in contact with living metabolites (Sarker et al., 2007). Another way of determining the MIC is by reading the optical density in a spectrophotometer, resulting in the absorbance of each well of the microplate.

Such tests can branch out into other different testing possibilities, such as the evaluation of extracts and compounds from natural products together with other antimicrobials against microorganisms, with the aim of verifying whether there is synergism or antagonism, as well as the inhibition of biofilm formation, among others. Table 2 summarizes the evaluated plant species, their antimicrobial activities against phytopathogens, the techniques applied, and the main findings. The selected studies, published between 2020 and 2024, are open-access articles from peer-reviewed journals with an impact factor above 3. These studies focus on plant-derived bioactive compounds tested against bacterial and fungal phytopathogens. The articles were retrieved from the Scopus database using various keyword combinations, including “phytopathogen and antimicrobial and plant and extract,” “plant and extract and fraction and phytopathogens,” “plant and extract and molecule and phytopathogen,” “plant and extract and isolated and compounds and phytopathogen,” and “phytopathogen and nanoparticles and antimicrobial.”

**Table 2:** Main techniques and methods for the antimicrobial evaluation of natural products against phytopathogens.

Plant	Phytopathogens evaluated	Extract or compounds	Techniques	Results	Reference
*Achillea millefolium* L., *Mentha piperita* L., *Salvia officinalis* L., *Equisetum arvense* L., *Urtica dioica* L., *Taraxacum officinale* (L.) Weber ex F. H. Wigg., *Elymus repens* (L.) Gould, *Hypericum perforatum* L., *Rosmarinus officinalis* Spenn., *Humulus lupulus* L., *Satureja hortensis* L., *Carum carvi* L., *Nigella sativa* L., *Thymus vulgaris* L., *Lavandula angustifolia* Mill., *Armoracia rusticana* G. Gaertn., B. Mey. and Scherb., *Allium sativum* L., *Syzygium aromaticum* (L.) Merr. and Perry, *Allium cepa* L., *Curcuma longa* L., *Polygonum bistorta* L. and *Polygonum aviculare*	*Colletotrichum coccodes*, *Phoma exigua*, *Fusarium sambucinum*, *Rhizoctonia solani*, *Alternaria tenuissima*, *Streptomyces scabiei*, *Pectobacterium carotovorum*, *Alternaria alternata*, *Alternaria solani*, *Fusarium oxysporum*	Water extracts, water-glycol extract, subcritical carbon dioxide extracts	Agar-well diffusion and agar-disc diffusion method and macro-broth dilution method	Inhibition zones between 1 and 59 mm for diffusion and with minimum inhibitory concentrations (MIC) ranging from approximately 0.9 to 25 mg/mL	Steglińska et al. (2022)
*33 Brassica oleracea* varieties	*Xanthomonas campestris, Agrobacterium rhizogenes, Fusarium oxysporum, Gibberella zeae, Phytophthora infestans*	Dichloromethane extract	Kirby–Bauer disk diffusion method and two-fold serial dilution method	The extract showed zones of inhibition of 15.90 and 28.60 mm and showed overall MICs in the range of 7.81 to 31.25 μg/mL	He et al. (2024)
*Gnaphalium uliginosum* L.	*Clavibacter michiganensis Erwinia carotovora* spp. *carotovora, Alternaria solani, Rhizoctonia solani*	Ethanolic extract	Agar diffusion and serial dilutions	MIC range of 2,500 to 78 μg/mL and inhibition halos of 24 to 13 mm	Davydova et al. (2024)
*Hibiscus rosa-sinensis*	*Xanthomonas oryzae pv. oryzae*	Cobalt oxide nanoparticles	Micro-dilution, Lesion length in rice plants after treatment and Biofilm inhibition	Biofilm inhibition up to 79,65% and inhibition zones ranging from 2,40 cm to 2.90 cm	Ogunyemi et al. (2023)
*Centaurea calcitrapa*	*Agrobacterium tumefaciens, Erwinia amylovora, Pseudomonas syringae* pv*. Aptata, Pseudomonas syringae* pv. *syringa, Xanthomonas campestris* pv*. campestris, Xanthomonas arboricola* pv. *juglandis*	Methanolic extract	Well diffusion test, Two-fold serial dilutions	Inhibition of 9.83 up to 30.5 mm, and MIC values ranging of 25–750 μg/mL	Dimkić et al. (2020)
*Camellia sinensis*	*Fusarium equiseti*	Zinc oxide nanoparticles	Inhibitory activity on agar	Growth Inhibition up to 84.8%	Subba et al. (2024)
*Atriplex glaucum and Calendula officinalis*	*Fusarium oxysporum, Colletotrichum gloeosporioides* and *Cladosporium cladosporioides,*	Methanolic extracts	Kirby–Bauer method (Disk diffusion)	Inhibitions halos up to 9.32 mm	Lazcano-Ramírez et al. (2023)
*Dunaliella salina*	*Pseudomonas syringae pv. tomato, Bacillus subtilis and Pectobacterium. carotovorum subsp. carotovorum*	Chloroform: Methanol extract, Ethanol extract and Hexane extract	Agar Disc Diffusion Method, broth dilution method and *in vivo* treatment evaluation in tomatoes	Inhibitions zone up to 20.0 mm and MICs of 0.3 mg/mL	Ambrico et al. (2020)
*Hedera helix*	*Saccharomyces cerevisiae* and *Diplodia corticola*	Aqueous Extract	Growth inhibition and Yeast Viability with the percentage of colony-forming units	Growth Inhibition up to 70% and percentage of colony-forming units of 0% in certain conditions	Crisóstomo et al. (2024)
*Erismadelphus exsul*	*Phytophthora infestans* and Zymoseptoria tritici	Ethanolic extract and fractions	Microplate serial dilution and mycelial growth inhibition test in microplates	Growth Inhibition up to 100%	Essono Mintsa et al. (2022)
*Moringa oleifera*	*Erwinia amylovora*	Methanolic, hydroalcoholic and hydroalcoholic with maltodextrins	Microdilution method and analysis of disease symptoms *in vivo*	Mic of >2 to 1 mg/mL, 80% reduction in biofilm formation, and reducing the wilting area by up to 80%	Fontana et al. (2022)
*Sambucus nigra*	*Diaporthe amygdali, Phytophthora megasperm* and *Verticillium dahliae*	Ammoniacal aqueous extract	Agar dilution method and *Ex Situ* protection assays on excised stems	Growth Inhibition up to 100% with a effective concentration of 50% at 193.9 μg/mL, and full protection of the stem at the concentration of 1875 μg/mL	Sánchez-Hernández et al. (2023)
*Artemisia annua* L*., Artemisia dracunculus* L*., Artemisia santonica* L*., Artemisia abrotanum* L. and *Artemisia scoparia* Waldst. and Kit	*Rathayibacter iranicus, Bacillus subtilis, Xanthomonas arboricola, Agrobacterium tumefacien, Alternaria solani, Fusarium graminearum* and *Rhizoctonia solani*	Ethanol extracts	Two-fold serial dilution and serial dilution method	MIC ranging of 310 to >5,000 μg/mL	Nikitin et al. (2023)
*Pavlova lutheri, Chaetoceros muelleri, Chlorella* sp., *Dunaliella tertiolecta, Haematococcus pluvialis, Isochrysis galbana, Nannochloropsis* sp., *Scenedesmus* sp., *Tetraselmis astigmatica, Tetraselmis chuii, Tetraselmis suecica, Limnothrix* sp. and *Spirulina* sp.	*Clavibacter michiganensis* and *Pseudomonas syringae*	Hexane, dichloromethane and methanol fractions	Disc-diffusion method, spot-on-lawn method and serial dilutions	MIC of 500 μg/mL to 1 mg/mL and inhibition halos up to 18.67 mm	Alsenani et al. (2020)
*Larrea nitida* Cav.	*Fusarium oxysporum, Fusarium verticillioides* and *Trichoderma harzianum*	Nanodispersions produced from the methanolic extract	Agar diffusion	Growth inhibition up to 48%	Rocha et al. (2023)
*Baccharis trinervis* Pers., *Baccharis prunifolia* Steyerm, *Baccharis zumbadorensis* Badillo	*Botrytis cinerea*	Methanol and dichloromethane extracts	Broth Microdilution Method and poisoned food technique	MIC ranging of 125 to 250 μg/mL, lowest IC50 of 3.1 μg/mL, an growth inhibition up to 72,05%	Pinto et al. (2022)
*Quercus ilex sub*sp. *ballota* (Desf.) Samp.	*Fusarium circinatum, Cryphonectria parasitica, Phytophthora cinnamomic,*	Aqueous ammonia extract	Agar dilution method and Protection Tests on Artificially Inoculated Excised Stems	EC_90_ values of 322, 295, and 75 μg/mL and achieving protection at of 782 μg/mL	Sánchez-Hernández et al. (2022)
*Capsicum annuum, Capsicum baccatum*	*Alternaria* sp.*, Aspergillus niger, Aspergillus dimorphicus, Fusarium oxysporum, Fusarium verticillioides, Penicillium citrinum* and *Rhizopus arrhizus*	Aqueous extracts	Well diffusion method, broth liquid dilution	Inhibition ratio up to 18%	Sepúlveda et al. (2024)
*Retama raetam*	*Stemphylium vesicarium*	Six compounds of the plant extract	Spot-inoculation and Inhibition of the fungal growth	Growth inhibition up to 55%	Soriano et al. (2022)
*Silybum marianum* (L.) Gaertn.	*Fusarium graminearum*	Peptides of the plant extract	Hyphal growth inhibition assays in microplate	Three peptides almost completely inhibited the hyphal growth	Fernández et al. (2021)
*Cassia fistula* (L.)	*Pseudomonas syringae, Fusarium oxysporum, Rhizoctonia solani, Sarocladium* sp.	Silver nanoparticles	Microplate antibiofilm assay, growth inhibition	Inhibitory zones of 12.2 mm and biofilm and fungal inhibition up to 78%	Danish et al. (2022)
*Ceropegia fusca, Argyranthemum broussonetii, Artemisia thuscula, Gymnosporia cassinoides, Cistus symphytifolius, Lavandula canariensis, Salvia canariensis, Apollonias barbujana sub*sp. *barbujana, Laurus novocanariensis, Ruta pinnata, Ruta chalepensis, Datura innoxia, Datura stramonium, Nicotiana glauca, Salpichroa origanifolia* and *Withania aristata.*	*Alternaria alternata, Botrytis cinerea,* and *Fusarium oxysporum*	Ethanolic plant extract	Mycelial growth inhibition by an agar-dilution method	Inhibition up to 72.22%	Reyes et al. (2022)
*Olive pomace*	*Xylella fastidiosa sub*sp. *Pauca, Pseudomonas syringae pv. tomato* and *Pectobacterium carotovorum* subsp. *carotovorum*	Phenolic extract	Disk diffusion assay, time-kill contact assay and Broth dilution assay	MIC ranging from 1.6 to 0.4 mg/mL	Greco et al. (2024)
*Alseis yucatanensis, Alvaradoa amorphoides, Annona primigenia, Bakeridesia notolophium, Bravaisia berlandieriana, Byrsonima bucidifolia, Calea jamaicensis, Cameraria latifolia, Chrysophyllum mexicanum, Coccoloba* sp.*, Croton arboreus, Croton itzaeus, Croton* sp.*, Cupania* sp.*, Diospyros* sp.*, Erythroxylum confusum, Erythroxylum rotundifolium, Erythroxylum* sp.*, Eugenia sp., Euphorbia armourii, Guettarda combsii, Helicteres baruensis, Heteropterys laurifolia, Hybanthus yucatanensis, Ipomoea clavata, Karwinskia humboldtiana, Licaria sp., Macroscepis diademata, Malpighia glabra, Morella cerifera, Mosannona depressa, Parathesis cubana, Paullinia sp., Piper neesianum, Psychotria sp., Randia aculeata, Serjania caracasana, Simarouba glauca, Stemmadenia donnell-smithii, Turnera aromatica*	*Fusarium equiseti* and *Fusarium oxysporum*	Aqueous Extracts, ethanolic Extracts and fractions	Broth microdilution	Lowest MIC of 1,000 μg/mL and growth inhibition up to 100%	Cruz-Cerino et al. (2020)
*Sideroxylon obtusifolium* and *Annona acutiflora*	*Thielaviopsis ethacetica*	Ethyl acetate and butanol extract	Hole diffusion test, Mycelial inhibition test and growth curve, Microdilution test	Inhibition halos up to 35.3 mm,and MIC of 25 to 6 mg/mL	Duarte et al. (2022)
*Monodora kerstingii*	*Fusarium oxysporum*	Crude extracts and fractions	Serial dilutions	MIC from >1,000 to 23 μg/mL	Fotso et al. (2020)
*Ptaeroxylon obliquum (Thunb.) Radlk*	*Aspergillus niger, Aspergillus parasiticus, Colletotrichum gloeosporioides, Fusarium oxysporum, Penicillium digitatum, Penicillium expansum, Penicillium italicum, Penicillium janthinellum, Rhizoctonia solani*	Acetone crude extracts, fractions, and isolated compounds	Serial microdilution assay	MIC from 1,250 to 32 μg/mL	Ramadwa et al. (2024)
*Capsicum chinense Jacq.,* var. ‘Habanero Mustard’, ‘Habanero Pastel’, ‘Trinidad Moruga Scorpion Red’, ‘Trinidad Moruga Scorpion Choco’, ‘Carolina Reaper’, ‘White Naga’, ‘Naga Morich Chocolate’	*Botrytis cinerea, Guignardia bidwellii, Plasmopara viticola*	Oleoresin	Diffusion technique, radial growth inhibition and Inhibition sporulation on leaf discs	Inhibition activity ranging from 0.001 to 12.5 mg/mL, with the complete inhibition in certain concentration	Vuerich et al. (2023)
*Cestrum nocturnum*	*Fusarium kuroshium, Fusarium solani*	Methanolic Crude Extract and fractions	Mycelial growth inhibition by microplate	Some fractions achieve 100% of inhibition	Valencia-Mejía et al. (2022)
*Zuccagnia punctata*	*Monilinia fructicola*	Ethanolic extract	Broth microdilution technique, e*x vivo* Antifungal Assay on Wounded Fruits and Cell Viability Assay with MTT	MIC from 250 to 62.5 μg/mL, and the treatments reduced the brown rot sporulation index compared to the control	Di Liberto et al. (2023)

According to Table 2, and according to database limitations, the most studied type of microorganism was fungi of the genus *Fusarium* sp. (16,8%). The most evaluated natural products regarding their antimicrobial activity were aqueous extracts (18.42%) and ethanolic extracts (15.79%).

Due to the lack of articles that establish clear benchmarks for promising antimicrobial activity against phytopathogens, this study proposes tentative threshold values to guide future evaluations. Values higher than 20.90 mm of inhibition halo are suggested as a indicative of promising activity. MIC values for bacteria of less than 2.5 mg/mL for the crude extract, 0.6 mg/mL for the fractions and 64 μg/mL for the compounds are proposed as benchmarks. In the case of the percentage inhibition of the growth of filamentous fungi, values of less than 52% inhibition may be considered promising.

**Figure 2:**
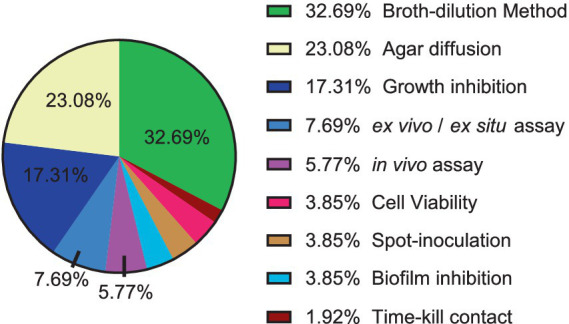
Distribution of techniques and methodologies for antibacterial and antifungal evaluation of natural products against phytopathogens.

Figure 2 illustrates the most commonly employed techniques and methods in the literature for assessing the activity of natural products against phytopathogenic microorganisms, along with their respective usage percentages.

The most used techniques in the articles for antimicrobial evaluation were dilution in broth and agar diffusion. The widespread of these techniques is due to their applicability, such as their use for solid or with difficult solubility substances, which allows the analysis of the growth of the microorganism in direct contact with the substance or compound, but there is less standardization and consequently variation between the results, in addition to the fact that the results can be difficult to interpret due to the interaction of the substance or compound with the agar. In the case of microdilution and macro-dilution in broth, the advantage is that it is well suited to samples that do not diffuse well in agar, such as for certain antibiotics (Satlin, 2019).

## 4 Recommended strategies, challenges and future directions

**Figure 3:**
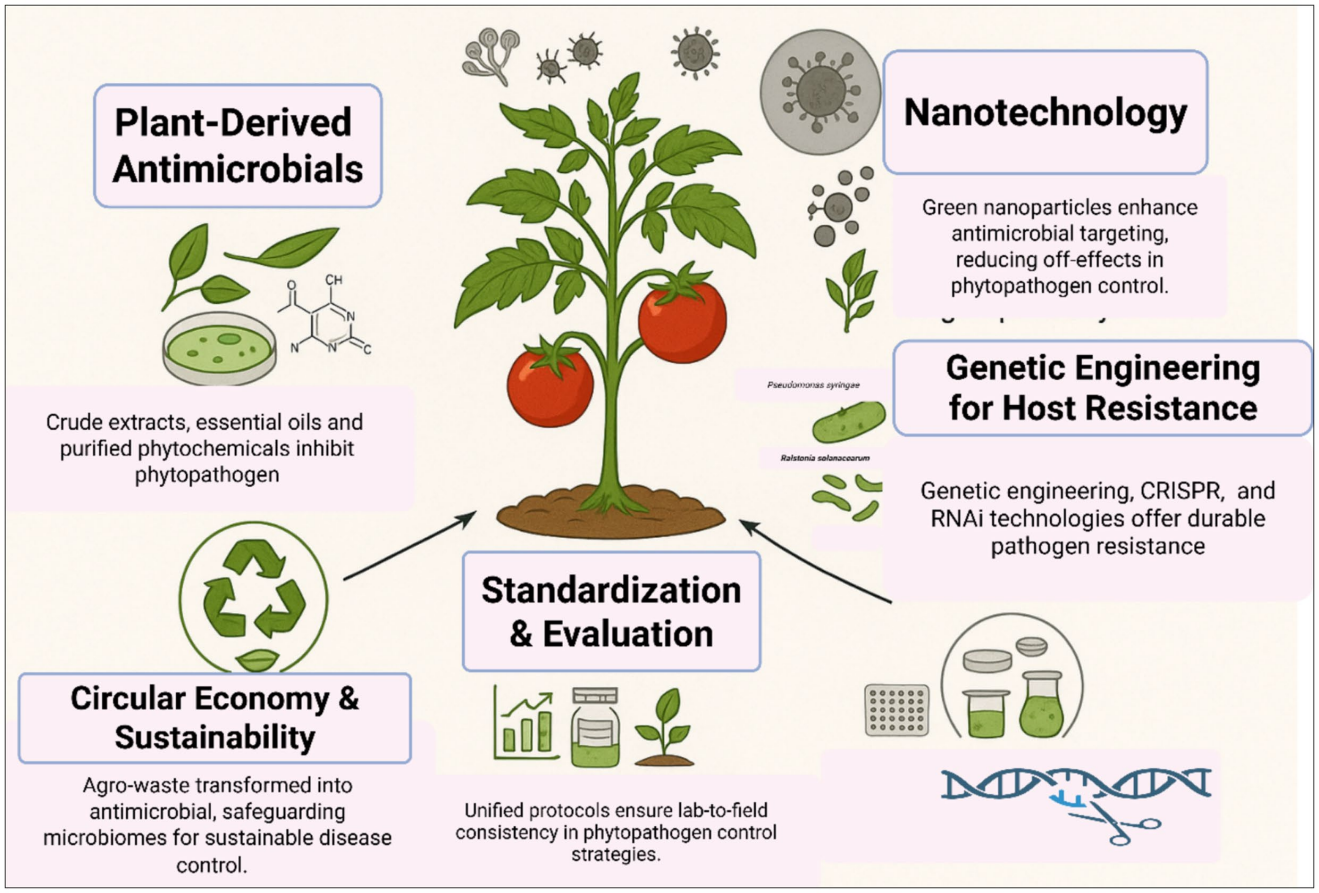
Multifaceted interventions for effective control of plant pathogens.

Effective phytopathogen control requires a multi-pronged strategy integrating phytochemical innovation, nanotechnology, host genetics, and ecological stewardship. Prioritizing standardized protocols, field validation, and socioeconomic feasibility will bridge the lab-to-field gap, advancing One Health-aligned agriculture (Figure 3). Plant-derived antimicrobials, including phytoalexins and phytoanticipins, represent a cornerstone of sustainable phytopathogen control. These compounds inhibit pathogens through mechanisms such as membrane disruption, enzyme inhibition, and interference with microbial signaling (González-Lamothe et al., 2009; Košćak et al., 2023). For instance, saponins like avenacin in oats and *α*-tomatine in tomatoes form complexes with fungal sterols, destabilizing membranes and suppressing infections (González-Lamothe et al., 2009). In addition to antagonistic bacteria and fungi, plant-derived bioactive compounds have emerged as promising biocontrol agents, demonstrating considerable efficacy in suppressing plant pathogen proliferation. These phytochemicals contribute to pathogen inhibition through direct antimicrobial activity, interference with virulence mechanisms, and the enhancement of host plant defense responses, thereby complementing microbial biocontrol strategies (da Silva et al., 2016; Najdabbasi et al., 2020). Moreover, the use of nanotechnology in plant disease diagnostics has transformational potential, enabling the creation of sophisticated instruments for the rapid and early identification of plant infections. Nanomaterials (1–100 nm) are well suited for this application owing to their superior surface-to-volume ratio and distinctive chemical, photonic, and electrical characteristics that fundamentally differ from those of bulk materials; the ability to execute precise molecular alterations, together with the unique optical properties of nanomaterials, enables ultrasensitive and effective pathogen detection systems (Ray et al., 2023). Genetic engineering and CRISPR-Cas9 genome editing provide precise, targeted alterations of plant genomes to improve resistance to phytopathogens. This method facilitates the insertion or deletion of certain genes, including pathogen-responsive R genes or susceptibility factors, while minimizing off-target effects, so expediting the creation of disease-resistant cultivars. The transfer of resistance-conferring genes across species expands the genetic repertoire beyond taxonomic boundaries, while robust integration of transgenes guarantees heritable resistance in vegetatively propagated crops, surpassing the constraints of traditional breeding methods. These solutions jointly enhance crop resilience by customized genetic modifications, reducing dependence on chemical interventions (Dong and Ronald, 2019; Gohil et al., 2024). Finally, the paradigm of plant disease management should shift from a narrow focus on crop yield to a comprehensive approach that integrates ecological sustainability, social acceptability, and economic feasibility. To achieve this, priority should be given to developing environmentally sustainable biocontrol agents, applied synergistically with other control measures under an integrated disease management system (He et al., 2021).

**Figure 4:**
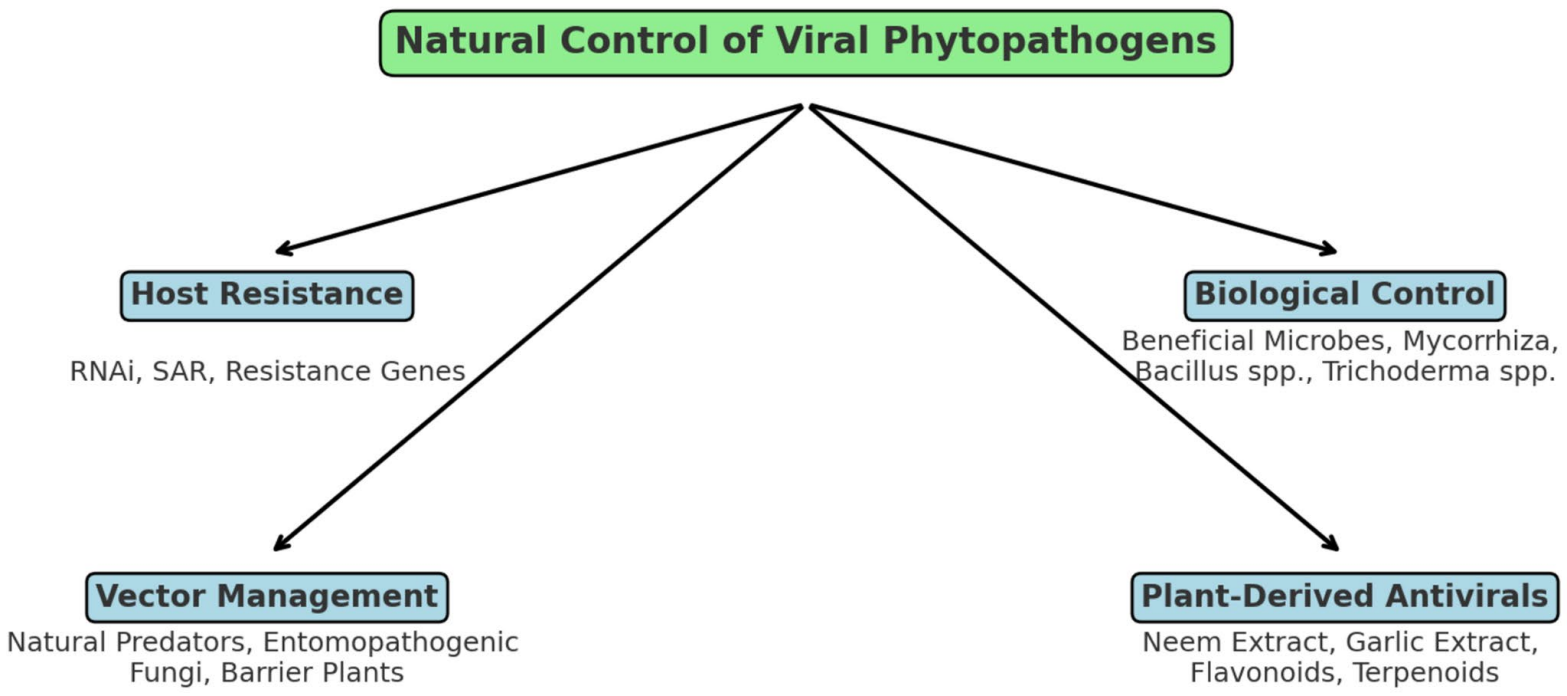
Schematic representation of natural control mechanisms against plant viral pathogens.

The control of plant viral diseases traditionally relies on chemical pesticides targeting insect vectors, cultural practices, and genetic resistance. However, natural control strategies offer an eco-friendly, sustainable alternative that minimizes environmental risks and enhances crop resilience (Figure 4). The central concept of natural control is connected to four main strategies: host resistance, biological control, vector management, and plant-derived antiviral compounds. Host resistance includes RNA interference and systemic acquired resistance. Vector management relies on natural predators and entomopathogenic fungi to limit insect-mediated virus transmission. Plant-derived antiviral compounds include flavonoids, terpenoids, and plant extracts such as neem and garlic, which interfere with viral replication and vector feeding behavior (Hudson, 2018; Jones, 2006).

The different methods used in the literature to evaluate the antimicrobial activity of natural products against phytopathogens make it difficult to compare results. An article may use the agar disk diffusion method and have a 20–28 mm growth inhibition halo as Mohamed et al. (2023) and another article a MIC result of 19.5 to 117 μg/mL as Makhubu et al. (2023). These two results cannot be compared due to the use of different principles, making it necessary to develop standard or common techniques, thus being able to compare extracts or compounds or nanoparticles in an easier, safer and standardized way.

Furthermore, there are few articles that address *in vivo* tests such as infection of the microorganism in the plant or fruit itself. Additionally, information on the epidemiology of diseases and global economic losses is scarce and what is available is outdated. There are few articles that explore the formation of biofilms of phytopathogens and the use of extracts and compounds with antibiofilm properties. This assessment is extremely important, since most microorganisms available in the environment are in the form of biofilms, distancing themselves from the real condition of infection (Ramey et al., 2004).

Key research gaps include elucidating the mechanisms of action of bioactive compounds, which could facilitate the design of more targeted and specific molecules. Additionally, evaluating these natural products directly on plants under varying soil types and climatic conditions, while considering their interactions with the soil microbiome, remains largely unexplored. There is also a pressing need for studies integrating antimicrobial assessments with emerging agricultural technologies, as well as comprehensive investigations into the toxicity and long-term environmental impact of these natural extracts on a broader scale.

## 5 Conclusion

The current review demonstrated that various natural products exhibit significant antimicrobial activity against phytopathogens, yielding promising and encouraging results. According to the literature, the most extensively evaluated natural products in terms of antiphytopathogenic activity were aqueous extracts and ethanolic extracts. The most frequently studied phytopathogens were *Fusarium* spp., while the predominant methodologies for antimicrobial assessment were dilution in broth and agar diffusion. Moreover, this review provides a comprehensive synthesis of existing studies to establish benchmarks for evaluating plant-derived antimicrobials against phytopathogens, offering practical criteria for prioritizing natural products in sustainable agriculture. Investigating the antimicrobial potential of natural extracts, fractions, and compounds against phytopathogens represents a promising avenue for their future application. A deeper understanding of their mechanisms of action may provide valuable insights into effective strategies for crop protection. Given the devastating impact of phytopathogens on agricultural productivity, advancing research in this field is crucial for the development of sustainable and efficient plant protection solutions.
